# A Bidirectional Design Method for Through-Glass Vias with Selective Laser Wet Etching Based on the Cross-Modal Learning Method

**DOI:** 10.3390/mi17010033

**Published:** 2025-12-27

**Authors:** Yongbo Meng, Liqing Wu, Bo Yuan, Xingping Zhou, Yan Li, Zhijun Zhang, Yuechun Shi

**Affiliations:** 1 Yongjiang Laboratory, Ningbo 315202, China; 2411100227@nbu.edu.cn (Y.M.); 20245239036@stu.suda.edu.cn (B.Y.); zhijun-zhang@ylab.ac.cn (Z.Z.); yuechun-shi@ylab.ac.cn (Y.S.); 2 Faculty of Electrical Engineering and Computer Science, Ningbo University, Ningbo 315211, China; liyan4@nbu.edu.cn; 3 Institute of Quantum Information and Technology, Nanjing University of Posts and Telecommunications, Nanjing 210003, China; zxp@njupt.edu.cn

**Keywords:** laser material processing, glass processing, selective laser etching, ultrafast optics, deep learning

## Abstract

As an interposer, Through-Glass Vias (TGVs) play a critical role in advanced packaging such as Co-packaged optics (CPO). Currently, due to the complex influence of laser wet-etching process parameters, the precise bidirectional prediction of TGV parameters and the etching morphology still remains a challenge. In this paper, a bidirectional design method for TGVs is proposed, which is based on the cross-modal learning method. By integrating a Cellular Automaton Etch-Diffusion (CAED) physical model with a Stable Diffusion (SD) architecture, accurate forward prediction from laser parameters to TGV morphology is realized successfully. In addition, the Contrastive Language–Image Pre-training (CLIP) model is also applied to achieve an efficient inverse design of TGVs. Furthermore, the generalization ability is examined in this paper, demonstrating significant robustness and stability of the generative model. The results provide an efficient method for enhancing TGV quality within a deep learning framework.

## 1. Introduction

To meet the demands of advanced three-dimensional packaging [1], heterogeneous integration has emerged as a critical pathway for enhancing integration density and device performance [2]. Due to the low electrical loss at high frequencies, relatively high stiffness, and adjustable thermal expansion coefficient of glass substrates, the Through-Glass Via (TGV) technique enables the fabrication and metallization of high-density, high-aspect-ratio glass vias, reducing device size while maintaining high-performance electrical interconnects [3,4,5]. The selective laser wet-etching (SLE) technique, with high fabrication precision, rapid processing, and low cost, has been an important means for TGV fabrication [6,7]. The SLE process is optimized through the adjustment of laser parameters and the implementation of various scanning strategies including segment scanning and contour scanning, based on a large number of experimental data [8,9]. In order to investigate the effects of laser parameters including spot radius and pulse energy on etching selectivity and key TGV morphological parameters such as aspect ratio, taper, and irregularity, the micro-crack-assisted etching model is developed to predict TGV morphology through the integration of laser processing, chemical etching, and substrate parameters [10]. A diffusion model is employed to explain the mechanism behind the high selectivity [9]. Moreover, some studies have established wet-etching models [5,11,12] and performed electromagnetic simulations on TGVs using HFSS software [5] to optimize the entire SLE process. Among them, the Cellular Automaton Etch-Diffusion (CAED) model [12] can be applicable to the complex chamfer geometries experimentally. In addition, the etching selectivity and diffusion coefficient, determined by fitting the etch depth to the etch time using the superdiffusion model, are used to predict the microchannel etch depth under specific laser and etching parameters for different etch times [13]. Since the SLE process involves multiple physical parameters such as laser pulse energy, repetition rate, and etchant concentration, the numerical simulation method is restricted in precisely determining the influence of each parameter and simultaneously adjusting multiple parameters. Existing studies have mainly optimized SLE-based TGV fabrication through extensive experimental parameter tuning and scanning strategies [6,7,8,9], and several physics-based models (e.g., micro-crack-assisted etching and diffusion-related wet-etching models) have been developed to explain the etching selectivity and to predict etching outcomes in a forward manner [5,9,10,11,12,13]. Currently, the main difficulties of SLE lie in the efficiency of visualizing the etching morphology in simulation, as well as in inversely deducing SLE processing parameters from etching morphology. In particular, there are few relevant reports on the inverse design for extracting laser parameters directly from the etching morphology. Therefore, it is necessary to develop an efficient method for the bidirectional design of TGVs.

The cross-modal learning framework possesses powerful perception and association capabilities for complex information by fusing heterogeneous data such as text and images [14,15]. Hence, this framework has been widely applied in multiple fields [16,17,18] to figure out synthesis problems. For instance, the video denoising diffusion model generates full-field stress evolution video frame sequences conditioned on stress–strain curves, aiding inverse design involving contact/buckling mechanisms in mechanical metamaterials [16]. Text encoders and cascaded diffusion models can achieve non-Abelian gauge field generation in circuit design, with a verification accuracy of 77.4% [17]. The Contrastive Language–Image Pre-training (CLIP) model successfully constructs a cross-modal semantic space between photonic crystal structural parameters and optical field distributions, while the Stable Diffusion (SD) model can generate optical fields from structural information [18]. Obviously, the cross-modal learning algorithm can provide a more efficient approach for complex physics models.

In this paper, a physics-based CAED simulator is combined with the cross-modal learning framework to enable bidirectional prediction of the SLE process. The CAED model is established with Super-Gaussian and Bessel-Gaussian beams of ultrafast laser, which separates the glass substrate into a cellular grid and describes the wet-etching evolution by local reaction–diffusion rules on the grid. The etching state of each cell is updated according to the etching selectivity and the neighboring cell states, thereby enabling an efficient simulation of morphology evolution during SLE wet etching. Based on this CAED method, the “SLE parameters–TGV morphology images” datasets are generated, providing physics-consistent supervision for training and evaluating the subsequent deep learning models. A cross-modal self-supervised learning framework is applied for the generation–inversion prediction task under the constraints of the SLE process. Under the constraints of the laser modification and wet-etching process, the SD architecture can achieve a forward design from SLE process parameters to TGV morphology images. Furthermore, using a CLIP algorithm it can establish a deep semantic alignment between TGV morphology images and SLE processing parameters, as well as enable the inverse extraction of laser parameters. Moreover, we further evaluate the model’s robustness and generalization across the parameter space to better characterize its applicability beyond the training distribution. The simulation results show that this work provides a new approach of intelligent design and optimization for the TGV fabrication process.

## 2. Principle

**Figure 1:**
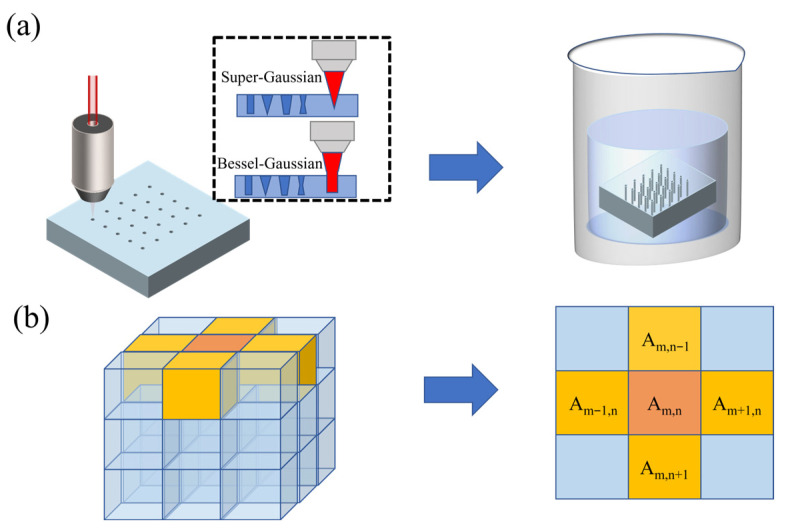
Schematic diagram of the SLE and modeling. (**a**) The target area on the glass substrate is modified using Super-Gaussian or Bessel-Gaussian beams, followed by wet etching of the modified region. (**b**) In the Cellular Automaton Etch-Diffusion model, the glass substrate is divided into several adjacent cells.

The SLE process can be divided into two major steps: laser-induced modification and wet etching. Figure 1a describes the principle of SLE, where a laser beam is illuminated on a glass substrate to create a modified area. Subsequently, after being corroded by the acidic etching solution, the modified area will exhibit higher etch selectivity than the unmodified area. Hence, by modulating the laser parameters, the modified area can be changed to obtain the desired TGV morphology. This paper employs the Super-Gaussian beams for layered scanning and the Bessel-Gaussian beams for single-pass penetration scanning to fabricate TGVs, respectively. Super-Gaussian beams can provide high selectivity and fine processing yet are limited in single-pulse processing ability. Conversely, Bessel-Gaussian beams allow efficient single-pulse modification at the expense of processing quality and selectivity. As shown in Figure 1a, the two types of laser beams are used to modify the glass.

A CAED model is employed to simulate the wet-etching process as shown in Figure 1b, which can be described by a set of two-dimensional reaction–diffusion equations [12] (as shown in Equation (1)). Equation (1) also can be extended to 3D. In order to facilitate the recognition of the SD model by the 2D image, only 2D models are considered here. In addition, the etching time is correlated with the third dimension (the z-direction) in the simulation with generality. Hence, the 2D TGV morphology in this paper is the cross-sectional view of the center of the 3D glass substrate after etching. In CAED model, the glass substrate can be regarded as a cuboid composed of multiple layers of two-dimensional planes, each of which is divided into *m* × *n* grids. The parameter *A_m,n_* is introduced to represent the glass concentration at the (*m*, *n*) grid. When *A_m,n_ =* 1 and *A_m,n_ =* 0, it signifies that the glass is unetched and completely etched, respectively. The initial concentration of the glass substrate remains constant. In the etching process, assuming that the chemical corrosion solution is uniformly diffused, the etch rate at grid point (*m*, *n*) is driven by the etch states of its four surrounding grid points (*m* − 1, *n*), (*m* + 1, *n*), (*m*, *n* − 1), (*m*, *n* + 1).

(1) $$\frac{1}{B}\frac{\partial }{\partial t}A(t,m,n)=\left(\frac{\partial^{2}}{\partial n^{2}}+\frac{\partial^{2}}{\partial m^{2}}\right)A(t,m,n)-\left(4+\frac{S(m,n)}{B}\right)\cdot (1-A(t,m,n))$$

(2) $$S_{m,n}=S_{0}\cdot \exp\left(-\frac{|n{|}^{N}}{|\omega_{n}{|}^{N}}-\frac{|m{|}^{N}}{|\omega_{m}{|}^{N}}\right)$$

(3) $$S_{m,n}=S_{0}\cdot \exp\left(\frac{-2r^{2}}{\omega^{2}}\right)\cdot {\left|J_{1}(k_{r}r)\right|}^{2}$$

In Equation (1), *B* represents the uniform etch rate of the chemical corrosion solution, and *A*(*t*, *m*, *n*) represents the glass concentration at position (*m*, *n*) at time *t*. *S_m_*_,*n*_ represents the etch selectivity of the modified region related to laser parameters, where the peak etch selectivity *S*_0_ can be equivalently expressed as the peak energy of the laser. The etch selectivity for Super-Gaussian and Bessel-Gaussian beams is defined as shown in Equation (2) and Equation (3), respectively. Where *ω* represents the beam waist radius, *N* is the Super-Gaussian exponent; *K_r_* is the radial wave vector; and *r* is the radial distance. When using the Super-Gaussian beam as the light source, the value of *S*_0_ is 10^6^. When using the Bessel beam as the light source, the value of *S*_0_ depends on the beam waist radius and pulse energy. The pulse duration of the laser is 300 fs, and the pulse energy is about 10 μJ.

**Figure 2:**
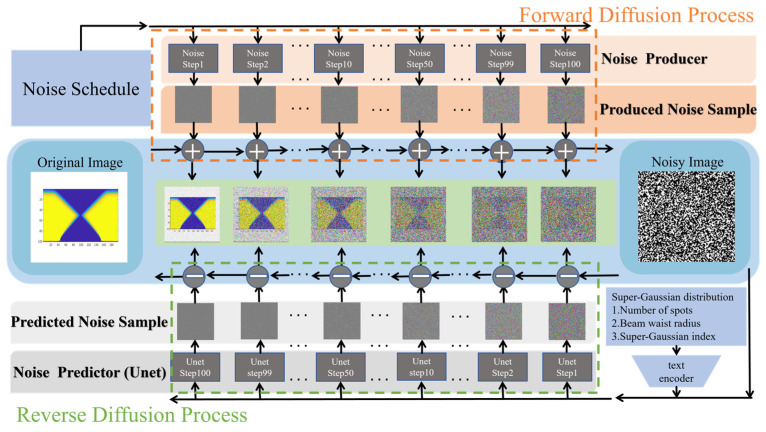
Schematic diagram of the forward diffusion and reverse diffusion in SD algorithm.

The SD algorithm can be used to build the text (laser parameters)-to-image (TGV morphology) model. Based on the datasets collected from the above CAED model, a denoising diffusion model [19] is used to generate TGV morphology images from SLE process parameters in SD algorithm. During model inference, a text encoder converts laser parameters into text embeddings, which can be recognized by the conditional image generation module. After sampling an initial noise, the U-Net network iteratively denoises under text guidance via Denoising Diffusion Implicit Models to restore the latent feature corresponding to the etching morphology. Subsequently, the decoder of the Variational Auto Encoder (VAE) outputs the TGV morphology image. During training, the text encoder first processes the laser parameters, while the VAE encoder compresses the TGV morphology image into a latent feature. A random timestep is then sampled, and noise is added to the latent feature. Leveraging cross-attention mechanisms, the U-Net network learns to predict the noise based on the SLE text embeddings, noisy TGV latent variables, and the timestep. The U-Net parameters are optimized by minimizing the mean squared error between the predicted noise and the true noise. Figure 2 illustrates the schematic of the forward and reverse diffusion processes. The forward diffusion process is a Markov Chain Process that gradually adds Gaussian noise to the original TGV image, transforming the initial image into a pure noise image over 100 steps. The reverse diffusion process is the inverse Markov Chain Process simulated by the U-Net network, which gradually denoises a sample from a pure Gaussian noise distribution to generate the target TGV morphology image. Using the same training framework, four hole type (straight hole, tapered hole, trapezoidal hole, and waist hole) datasets are mixed as the training datasets for the SD model. It is worth noting that, although the training framework is fully general, for different hole types, the generalization ability, inference efficiency, and attainable image quality of the SD model will vary. Hence, in the subsequent result analysis, three evaluation metrics are employed to assess the training effects of different hole types, and the Gaussian Process Regression (GPR) model is used to calculate the generalization ability. In the training process, the datasets of straight hole, tapered hole, and trapezoidal hole each consist of 8000 images, while the relatively complex waist hole dataset contains 10,000 images. In addition, the proportion of the training set, validation set, and test set in the SD model is 7:2:1.

**Figure 3:**
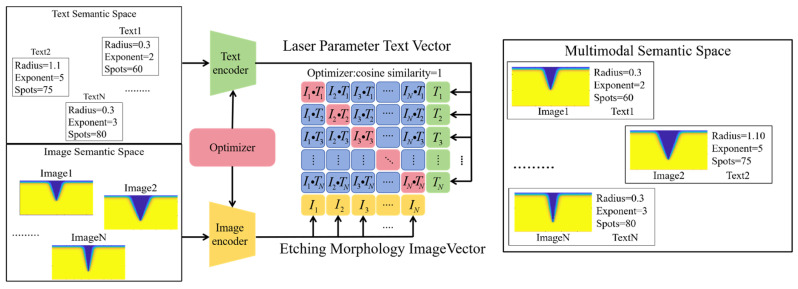
Schematic diagram of the CLIP algorithm. The optimizer achieves precise matching and prediction between SLE text feature vectors and TGV image feature vectors by maximizing the cosine similarity.

The CLIP contrastive learning method [20] is used to map TGV morphology images and SLE processing parameters into the same embedding space. Through contrastive learning, the embedding distance is minimized for matched morphology images and SLE parameters, while the distance is maximized for mismatched pairs. The schematic diagram of the CLIP model is shown in Figure 3. For the Super-Gaussian beam, the SLE etching capability is determined by three key parameters: the number of pulses, the beam waist radius, and the Super-Gaussian exponent, which are selected as the input text. In the cross-modal space, association and matching of cross-modal information are achieved by computing the cosine similarity between pairwise SLE parameter text vectors *I* and TGV morphology image vectors *T*. The cosine similarity formula is shown in Equation (4), where ||I|| represents the text vector norm. When similarity (*I*, *T*) equals 1, it indicates that the TGV morphology image and SLE process parameters pair are close in the semantic space, meaning they are a perfect match.

(4) $$similarity(I,T)=\frac{I\cdot T}{\left||I||\times ||T|\right|}$$

## 3. Results and Analysis

**Figure 4:**
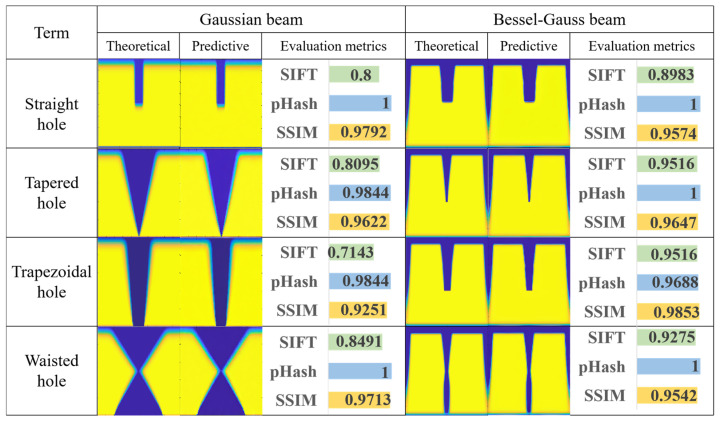
The stable diffusion model generation results are displayed in a quality evaluation chart. TGVs of various types, including straight holes, tapered holes, trapezoidal holes, and waist holes, are generated using SLE text parameters based on Super-Gaussian and Bessel-Gaussian beams. The theoretical TGV images and the generated TGV images are evaluated using SIFT, pHash, and SSIM evaluation metrics.

To assess the reliability and accuracy of the TGV morphology images generated by the SD model, three evaluation metrics are used in this paper: Scale-Invariant Feature Transform (SIFT) [21], Perceptual Hash (pHash) [22], and Structural Similarity Index (SSIM) [23], as shown in Figure 4. These metrics capture different physically meaningful aspects of agreement between the SD-generated morphology and the CAED reference. SIFT is feature- and edge-sensitive because it compares local feature points; SSIM is structure-sensitive because it jointly evaluates luminance, contrast, and structural consistency; and pHash emphasizes global perceptual similarity by comparing low-frequency content. The SIFT similarity is computed by matching feature points using a nearest-neighbor distance ratio criterion. The similarity score is defined as the fraction of matched feature points (Equation (5)), where *N*_ref_ and *N*_gen_ denote the numbers of feature points extracted from the reference and generated images, respectively, and *N*_match_ denotes the number of matched feature points. SSIM measures structural similarity between two images (Equation (6)). In the SSIM formulation, $\mu_{x}$ and $\mu_{y}$ are the mean intensities of images x and y; $\sigma_{x}^{2}$ and $\sigma_{y}^{2}$ are their variances; and $\sigma_{xy}$ is the covariance between x and y. The pHash evaluates perceptual similarity by converting images to grayscale, extracting low-frequency components, and constructing a compact hash representation. As shown in Equation (7), the low-frequency coefficient *F*(*i*, *j*) at pixel position (*i*, *j*) is used to compute the average over the low-frequency region, where *W* and *H* denote the image width and height, respectively.

(5) $$Similarity=\frac{N_{\mathrm{match}}}{\min(N_{\mathrm{ref}},N_{\mathrm{gen}})}$$

(6) $$\mathrm{SSIM}(x,y)=\frac{\left(2\mu_{x}\mu_{y}+C_{1})(2\sigma_{xy}+C_{2}\right)}{\left(\mu_{x}^{2}+\mu_{y}^{2}+C_{1})(\sigma_{x}^{2}+\sigma_{y}^{2}+C_{2}\right)}$$

(7) $$\mu =\frac{1}{W\times H}\sum_{i=1}^{H}\sum_{j=1}^{W}F_{\mathrm{low}}(i,j)$$

It is found that the results using Bessel-Gaussian beams generally surpass those using Super-Gaussian beams. The fundamental reason lies in the unique energy distribution of Bessel-Gaussian beams containing high-intensity diffraction rings, causing the edge light intensity to be significantly higher than the modification threshold during laser etching of glass. As a result, the material undergoes complete modification and forms sharp boundaries in the CAED model simulation process. In contrast, Super-Gaussian beams exhibit insufficient edge light intensity, leading to a gradual modification transition zone along the boundary of the TGV morphology. The SD model struggles to accurately generate the gradual edge details anticipated by Super-Gaussian simulations. Due to high sensitivity to local features (especially edges), the SIFT can effectively distinguish the edge differences between the simulations using the CAED model and generations from the SD model. Additionally, Bessel-Gaussian simulations require fewer input parameters, reducing the learning difficulty for the SD model and enhancing generation quality.

**Figure 5:**
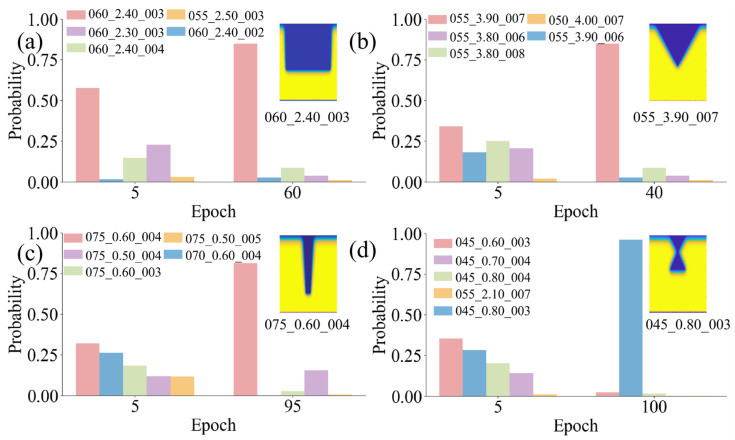
CLIP model prediction results for SLE parameters. The most probable SLE parameters are inversely derived by inputting TGV morphology images. In the legend, the SLE process parameters are set as pulse number_beam waist radius_Super-Gaussian exponent. (**a**), (**b**), (**c**), and (**d**) describe the process of reversely extracting laser parameters from the etching morphologies of straight holes, tapered holes, trapezoidal holes, and waist holes, respectively.

As shown in Figure 5, four sets of TGV morphology images with different SLE process parameters are input to the fine-tuned CLIP model for TGV image–SLE text matching test. Four types of TGV structures are fabricated with distinct SLE parameters (pulse count, beam waist radius, and Super-Gaussian exponent). In Figure 5, an epoch refers to one full pass of the CLIP training procedure over the entire training datasets. Specifically, the CLIP model processes all training image–text pairs once via multiple mini-batches and updates parameters through backpropagation and gradient-based optimization. Then it performs inference with the updated parameters to obtain the matching probabilities, which represents the degree of matching between the text and the image. Figure 5a shows the process of extracting laser parameters such as a pulse count of 60, a Gaussian beam waist radius of 2.40 μm, and a Gaussian exponent of 3 from the straight hole morphology image. Figure 5b illustrates the extraction of laser parameters, including a pulse count of 55, a Gaussian beam waist radius of 3.90 μm, and a Gaussian exponent of 7, from the tapered hole morphology image. Similarly, Figure 5c,d present the reverse extraction processes of laser parameters for the trapezoidal hole and waist hole, respectively. Additionally, an interfering factor is introduced by inputting a set of text descriptions similar to the actual SLE process parameters. The results show that, in the early training stages, the model shows the prediction probability for all candidate SLE parameters. As training epochs increase, the probability of mismatched parameters gradually converges to 0, while the probability of matched parameters converges to 1. Hence, an inverse prediction model is successfully set up to estimate SLE process parameters from TGV morphology images. As shown in Figure 5a,b, after 40 and 60 training epochs, the predictions for both tapered and straight holes gradually approach the actual values. While more-complex waist holes and trapezoidal holes require about 100 epochs to reach a stable state. The results can well demonstrate the inverse design capability of proposed CLIP model for TGVs.

Furthermore, in order to evaluate the generalization ability of the established model on untrained data, a Gaussian Process Regression model based on Bayesian optimization [24] is constructed. The input features of the GPR model are described by a three-dimensional parameter vector X: Super-Gaussian exponent *N*, beam waist radius *ω*_0_, hole-type number *type*. The output target *y* is the SSIM ranging from 0 to 1. The SSIM value represents the degree of similarity between the etching morphology generated by the model and the ideal morphology, signifying the generalization ability. The kernel function hyper-parameters *θ* are globally optimized via Bayesian optimization, with the objective function being the minimization of the negative log marginal likelihood: L(θ)=−logp(y|X,θ). In the optimization process, it reaches the global optimum at the 18th iteration. The final solved parameters in the optimization are as follows: signal variance *Ϭ_f_*^2^ = 3.3718; characteristic length scales *ℓ*_N_ = 1.7400, *ℓ*_ω0_ = 2.8285, *ℓ*_type_ = 0.0266; noise standard deviation *Ϭ*_n_ = 0.0126; basis function coefficient β = [0.9717]; bias term as a constant. The goodness-of-fit (R^2^) is equal to 0.983, indicating that the model accurately captures the nonlinear relationship between SSIM and SLE process parameters. The very small length scale for hole type suggests a strong influence of hole type on SSIM, where slight changes in hole shape cause significant output fluctuations. In contrast, the beam waist radius has the largest length scale value, indicating that SSIM changes smoothly with radius, and SD has strong robustness to radius changes. The trained GPR model predicts the SSIM, computes its 95% confidence interval, and quantifies the distance between test points and the training distribution with the Mahalanobis distance [25].

**Figure 6:**
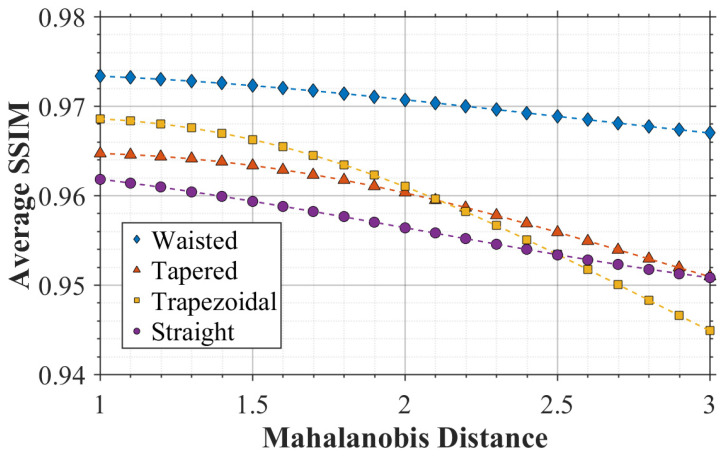
SD model generalization ability test results. Model generalization capability weakens as the Mahalanobis distance from the training dataset increases.

Figure 6 shows the distribution of generalization ability within the untrained parameter space. To quantify the deviation of the testing parameter and the training data, the Mahalanobis distance is introduced as a covariance-aware measure of distribution shift in the joint parameter space. Each sample is represented by the parameter vector $x={\left[\;N,\;\omega_{0},\;type\;\right]}^{T}$, where N is the Super-Gaussian exponent; $\omega_{0}$ is the beam waist radius; and type denotes the hole-type index. Using all training samples $\{x_{i}{\}}_{i=1}^{M}$, the mean vector $\mu =\frac{1}{M}\sum_{i=1}^{M}x_{i}$ and covariance matrix $\Sigma =\frac{1}{M-1}\sum_{i=1}^{M}\left(x_{i}-\mu \right){\left(x_{i}-\mu \right)}^{T}$ are computed, respectively. The Mahalanobis distance of a test sample is then defined as $D_{M}\left(x\right)={\left(x-\mu \right)}^{T}\Sigma^{-1}\left(x-\mu \right)$. Compared with Euclidean distance, $D_{M}\left(x\right)$ accounts for differences in parameter scales and correlations and thus provides a more appropriate indicator of distribution shift. A small value of $D_{M}$ indicates that the test condition lies within (or close to) the high-density region covered by the training data, whereas a large value of $D_{M}$ corresponds to the out-of-distribution case. Although all hole types maintain high fidelity, the generalization capability of SD model shows a decaying trend. As the Mahalanobis distance gradually increases, differences among hole types emerge: trapezoidal holes exhibit severe decay, while waist holes maintain optimal robustness. The generalization capabilities of hole type mainly rely on the training data distribution—waist hole samples constitute a larger proportion of the total dataset, leading the model to learn more thoroughly about common hole types that frequently appear in the dataset.

## 4. Discussion

The proposed method in this paper effectively leverages physical priors from the CAED model to enhance the accuracy and efficiency of both forward prediction and inverse design of TGVs. The results demonstrate that this method maintains high-quality performance across various TGV types, with its robustness closely related to the training data distribution. Notably, for structures that are well represented in the training set, such as waist holes, the established model exhibits better robustness.

Under the current conditions of small-scale datasets, the predictive capability of model gradually weakens as the Mahalanobis distance between test samples and the training distribution increases. This limitation can be alleviated by expanding the size of the dataset and strengthening the physical constraints. On the other hand, to better align with the actual TGV manufacturing process, this study can further expand the range of process parameters (such as etching time, etching solution concentration and temperature, and etching solution spray direction) to establish a complete process–morphology mapping relationship. By incorporating these parameters into the text–image cross-modal learning framework, not only can the microscopic mechanism of TGV formation under the coupling of multiple physical fields be revealed, but also the prediction accuracy and process guidance value of the model in real industrial scenarios can be enhanced.

In addition, although the current model already involves the basic structures of TGVs such as straight holes, tapered holes, trapezoidal holes, and waist holes, it should be extended to accommodate more-complex geometric morphologies. In the practical application of advanced packaging, it often also contains complex non-standard hole structures (such as bell-shaped holes, asymmetric holes, multi-step holes, and even three-dimensional curved micro-channels). This can further enhance the model’s recognition and generation capabilities, thereby enabling the generation of more-complex TGV geometric forms. This anisotropic hole structure has unique advantages in aspects such as high-frequency signal transmission and thermal stress matching, but the coupling of laser modification and etching dynamics of the physical model is more complex than simple hole structures. The physical model used in this paper is an approximate SLE simulation model including two processes: laser-induced modification and chemical corrosion, while the thermal accumulation, reactant accumulation, and femtosecond filamentation effects during the wet-etching process in the experiment were not fully considered. Therefore, in subsequent research, to enhance the model’s adaptability to diverse TGV morphologies and the flexibility of inverse design, it is necessary to combine multi-physics field simulation models, cross-modal deep learning, and reinforcement learning algorithms. This is expected to support the future development of the TGV technique towards functionalization and customization.

Furthermore, the aforementioned technique has certain requirements for the input dimensions and complexity of the AI algorithm. To address this challenge, advanced architectures such as attention mechanisms and graph neural networks could be introduced to enhance the model’s ability in capturing the implicit relationships between high-dimensional parameters of SLE processes and complex morphologies of TGVs. Meanwhile, a multi-task learning strategy could enable the simultaneous output of key quality indicators (such as surface roughness and hole diameter error), thereby providing more-comprehensive decision support for process optimization.

## 5. Conclusions

This paper introduces a cross-modal deep learning framework into the physical model of selective laser etching to establish the bidirectional design of TGV structures. A forward model based on a Stable Diffusion architecture is proposed to generate TGV morphology from process parameters and an inverse model is constructed using a CLIP algorithm to extract parameters from the target morphology. In addition, the established modal has been proven by the Gaussian Process Regression algorithm to have high accuracy and robust generalization across various TGV types. The approach in this paper provides a novel, efficient pathway for the intelligent design and optimization of TGVs and potentially other complex fabrication processes in Co-packaged optics.

## Data Availability

The raw data supporting the conclusions of this article will be made available by the authors on request.
